# Protein arginine methyltransferase 1 (PRMT1) represses MHC II transcription in macrophages by methylating CIITA

**DOI:** 10.1038/srep40531

**Published:** 2017-01-17

**Authors:** Zhiwen Fan, Jianfei Li, Ping Li, Qing Ye, Huihui Xu, Xiaoyan Wu, Yong Xu

**Affiliations:** 1 Department of Pathology, Nanjing Drum Tower Hospital, Affiliated Hospital to Nanjing University, Nanjing, China; 2 Key Laboratory of Cardiovascular Disease, Department of Pathophysiology, Nanjing Medical University, Nanjing, China; 3 Department of Gastroenterology, Second Hospital Affiliated to Nanjing Medical University, Nanjing, China

## Abstract

Efficient presentation of alien antigens triggers activation of T lymphocytes and robust host defense against invading pathogens. This pathophysiological process relies on the expression of major histocompatibility complex (MHC) molecules in antigen presenting cells such as macrophages. Aberrant MHC II transactivation plays a crucial role in the pathogenesis of atherosclerosis. Class II transactivator (CIITA) mediates MHC II induction by interferon gamma (IFN-γ). CIITA activity can be fine-tuned at the post-translational level, but the mechanisms are not fully appreciated. We investigated the role of protein arginine methyltransferase 1 (PRMT1) in this process. We report here that CIITA interacted with PRMT1. IFN-γ treatment down-regulated PRMT1 expression and attenuated PRMT1 binding on the MHC II promoter. Over-expression of PRMT1 repressed MHC II promoter activity while PRMT1 depletion enhanced MHC II transactivation. Mechanistically, PRMT1 methylated CIITA and promoted CIITA degradation. Therefore, our data reveal a previously unrecognized role for PRMT1 in suppressing CIITA-mediated MHC II transactivation.

T lymphocytes dependent immunity plays an important role in host defense by eliminating invading pathogens^1^. Inadequate levels of circulating T lymphocytes are associated with such human pathologies as acquired immune deficiency syndrome (AIDS) and bare lymphocyte syndrome (BLS)^2^. On the other hand, excessive activation of T lymphocytes results in chronic inflammation and highlights a host of cardiovascular and metabolic diseases including atherosclerosis^3^,^4^,^5^. Therefore, understanding the molecular mechanism that contributes to the regulation of T lymphocytes may help develop novel therapeutic solutions.

Antigen presentation by antigen presenting cells (APCs) represents a key step in T lymphocyte activation, a pathophysiological process that depends on the expression of class II major histocompatibility complex (MHC II) molecules^6^,^7^. MHC II is constitutively expressed in certain types of APCs (e.g., dendritic cells), but can be induced in other APCs (e.g., macrophages) by the pro-inflammatory cytokine interferon gamma (IFN-γ) at the transcriptional level^8^. MHC II transactivation by IFN-γ relies on the formation of a multi-protein enhanceosome, of which class II transactivator (CIITA) constitutes a core component, on the MHC II gene promoters^9^. Whereas CIITA loss-of-function mutations leads to ineffectual MHC II transactivation and immunodeficiency, CIITA hyperactivation is associated with aberrant MHC II transactivation and chronic inflammation^10^. CIITA activity can be modulated at both transcriptional and post-translational levels^11^. Previously we have shown that several different post-translational modifications contribute to differential modulation of CIITA activity. For instance, histone deacetylase 2 (HDAC2) mediated deacetylation of CIITA targets CIITA for proteasomal degradation and attenuates MHC II transactivation^12^. On the contrary, SIRT1 mediated deacetylation of CIITA enhances CIITA stability and promotes MHC II transactivation^13^.

Protein arginine methyltransferase 1 (PRMT1) belongs to the family of proteins that specialize in modifying arginine residues of both histones and non-histone factors^14^. Mounting evidence has suggested that PRMT1 is intimately involved in the immune response. For instance, Browne *et al*. have shown that PRMT1 inhibition attenuates the expression of HLA-A, but curiously not HLA-E, by IFN-γ in cancer cells^15^. PRMT1 has also been shown to repress NF-κB-mediated inflammation by methylating and preventing RelA from binding to target genes^16^. Likewise, PRMT1-mediated methylation of TNF receptor-associated factor 6 (TRAF6), an integral part of the signalosome that activates NF-κB, also represses inflammation^17^. In the present study we investigated the involvement of PRMT1 in IFN-γ-induced, CIITA-dependent MHC II transactivation in macrophages. Our data demonstrate that PRMT1 represses MHC II transcription in macrophages by methylating CIITA and promoting CIITA degradation.

## Results

### PRMT1 interacts with CIITA

**Figure 1:**
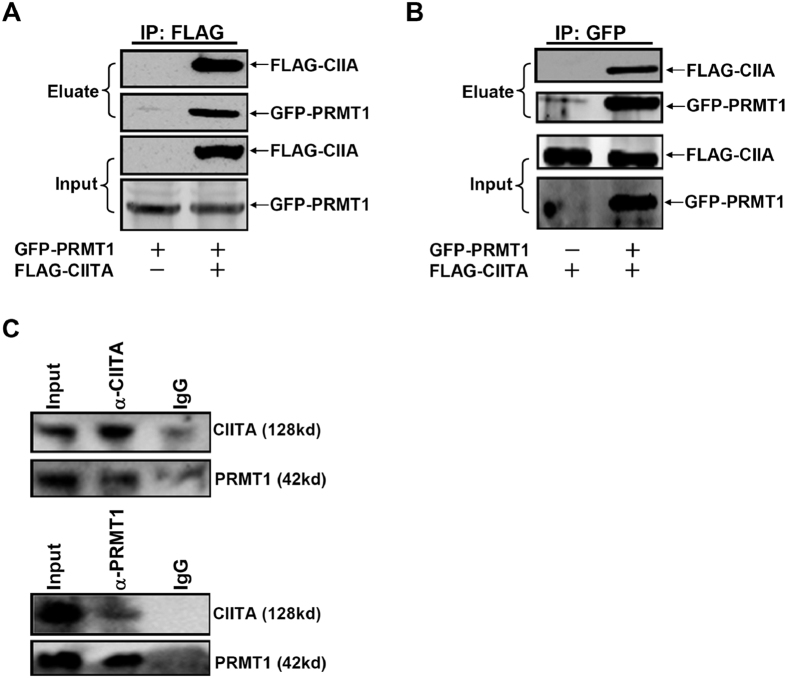
PRMT1 interacts with CIITA. (**A**) HEK293 cells were transfected with indicated expression constructs. Immunoprecipitation was performed with anti-FLAG and Western blotting was performed with anti-FLAG or anti-GFP. (**B**) HEK293 cells were transfected with indicated expression constructs. Immunoprecipitation was performed with anti-GFP and Western blotting was performed with anti-FLAG or anti-GFP. (**C**) RAW264 cells were treated with IFN-γ for 24 hours. Whole cell lysates were immunoprecipitated with anti-CIITA, anti-PRMT1, or a control IgG.

In order to determine whether PRMT1 could interact with CIITA, we performed co-immunoprecipitation experiments. To this end, GFP-tagged PRMT1 construct was transfected into HEK293 cells with or without FLAG-tagged CIITA construct. Anti-FLAG antibody precipitated PRMT1 only when FLAG-CIITA was present (Fig. 1A). The interaction between PRMT1 and CIITA was further corroborated by a reciprocal co-immunoprecipitation experiment in which FLAG-tagged CIITA construct was transfected into HEK293 cells with or without GFP-tagged PRMT1 construct: anti-GFP antibody precipitated CIITA only when GFP-PRMT1 was present (Fig. 1B). Importantly, we were able to confirm the interaction between endogenous CIITA and PRMT1 in mouse macrophage cells (RAW264): an anti-CIITA antibody precipitated PRMT1 while an anti-PRMT1 antibody precipitated CITIA (Fig. 1C). Together, these data suggest that PRMT1 could form a complex with CIITA in cells.

### IFN-γ down-regulates PRMT1 expression and activity in macrophages

**Figure 2:**
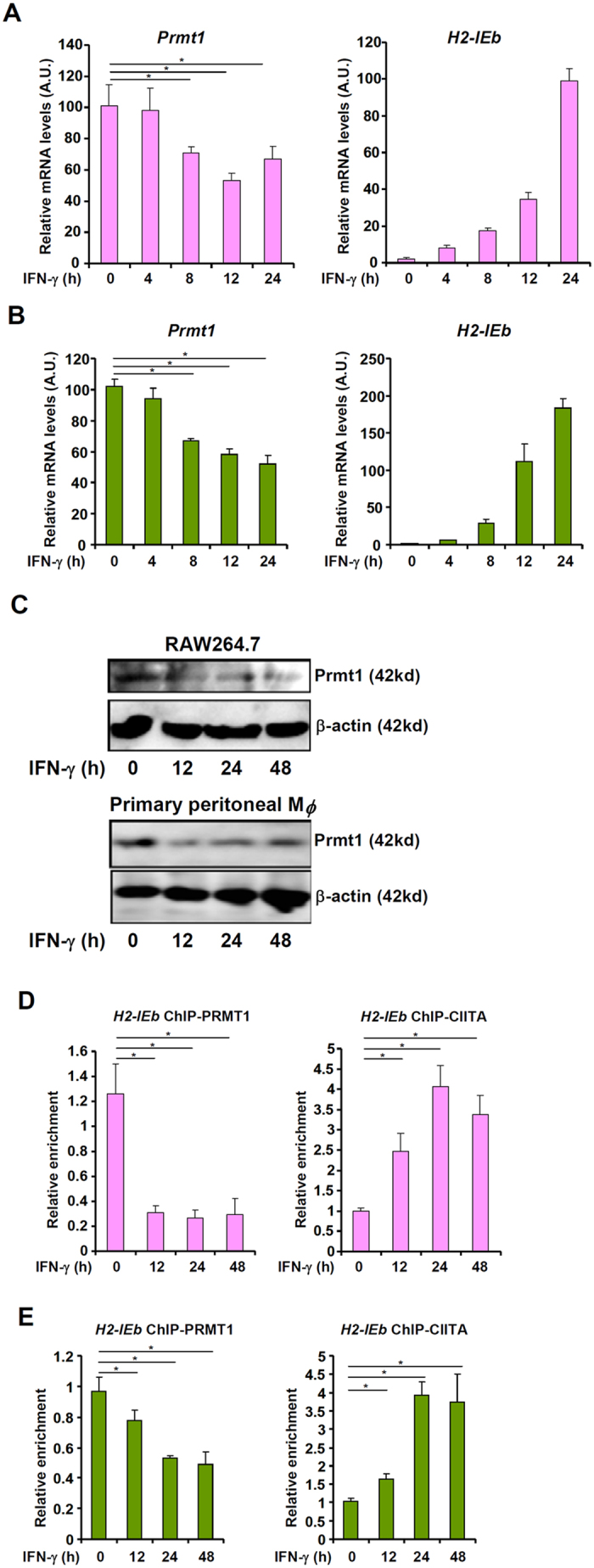
IFN-γ suppresses PRMT1 in macrophages. (**A,B**) RAW264 cells (**A**) or mouse primary peritoneal macrophages (**B**) were treated with IFN-γ and harvested at indicated time points. PRMT1 mRNA levels were evaluated by qPCR. (**C**) RAW264 cells or mouse primary peritoneal macrophages were treated with IFN-γ and harvested at indicated time points. PRMT1 protein levels were evaluated by Western blotting. (**D,E**) RAW264 cells (**D**) or mouse primary peritoneal macrophages (**E**) were treated with IFN-γ and harvested at indicated time points. PRMT1 binding to the MHC II gene promoter was examined by ChIP.

IFN-γ is the most potent stimulator of MHC II transcription in macrophages^8^. Therefore, we next examined the effect of IFN-γ treatment on PRMT1 in RAW264 cells. As shown in Fig. 2A and B, accompanying induction of MHC II (H2-IEb) molecule there was a simultaneous down-regulation of PRMT1 messages by IFN-γ as early as 8 hours following treatment in RAW264 cells and primary mouse peritoneal macrophages. Similarly, Western blotting analysis revealed that IFN-γ treatment also suppressed PRMT1 protein levels (Fig. 2C). Finally, chromatin immunoprecipitation (ChIP) assay showed that IFN-γ treatment decreased PRMT1 occupancies on the MHC II promoter paralleling an increase in CIITA binding on the same site in RAW264 cells (Fig. 2D) and in primary mouse peritoneal macrophages (Fig. 2E). Collectively, these data suggest that IFN-γ treatment may have a negative impact on PRMT1 expression and activity in macrophages.

### PRMT1 represses MHC II transcription in macrophages

**Figure 3:**
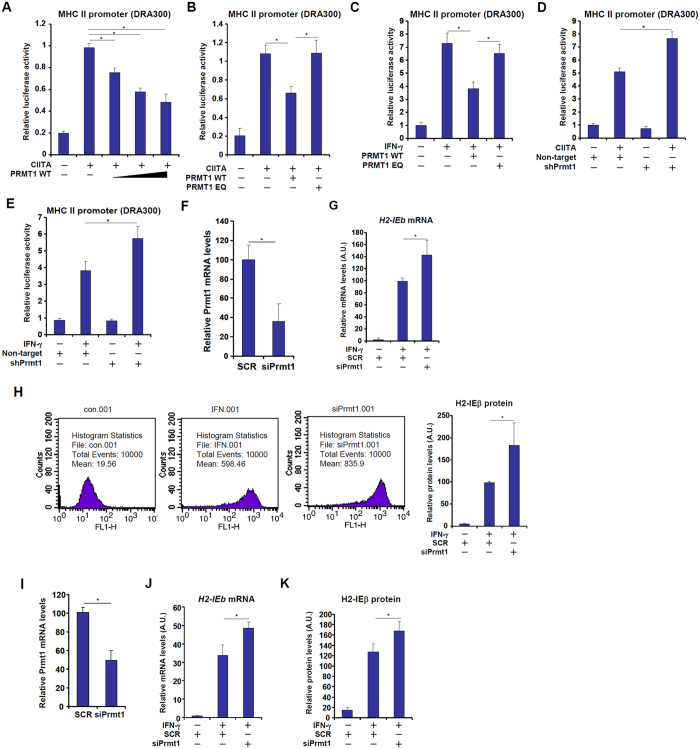
PRMT1 represses MHC II transcription in macrophages. (**A,B**) An MHC II promoter-luciferase construct (DRA300) was transfected into RAW264 cells with indicated plasmids. Luciferase activities were normalized to protein concentration and GFP fluorescence for transfection efficiency and expressed as relative luciferase activity compared to the control group. (**C**) An MHC II promoter-luciferase construct (DRA300) was transfected into RAW264 cells with indicated plasmids followed by treatment with IFN-γ. Luciferase activities were normalized to protein concentration and GFP fluorescence for transfection efficiency and expressed as relative luciferase activity compared to the control group. (**D**) An MHC II promoter-luciferase construct (DRA300) was transfected into RAW264 cells with CIITA and/or shRNA plasmid targeting PRMT1. Luciferase activities were normalized to protein concentration and GFP fluorescence for transfection efficiency and expressed as relative luciferase activity compared to the control group. (**E**) An MHC II promoter-luciferase construct (DRA300) was transfected into RAW264 cells with or without an shRNA plasmid targeting PRMT1 followed by treatment with IFN-γ. Luciferase activities were normalized to protein concentration and GFP fluorescence for transfection efficiency and expressed as relative luciferase activity compared to the control group. (**F**) RAW264 cells were transfected with siRNA targeting PRMT1 or a random siRNA (SCR). Knockdown efficiency was verified by qPCR. (**G,H**) RAW264 cells were transfected with siRNA targeting PRMT1 or a random siRNA (SCR) followed by treatment with IFN-γ. MHC II expression was examined by qPCR (**G**) and flow cytometry (**H**). (**I**) Mouse primary peritoneal macrophages were transfected with siRNA targeting PRMT1 or a random siRNA (SCR). Knockdown efficiency was verified by qPCR. (**J, K**) Mouse primary peritoneal macrophages were transfected with siRNA targeting PRMT1 or a random siRNA (SCR) followed by treatment with IFN-γ. MHC II expression was examined by qPCR (J) and flow cytometry (**K**).

Now that we observed a down-regulation of PRMT1 expression and activity by IFN-γ, we hypothesized that PRMT1 might repress MHC II transcription in macrophages. To verify this hypothesis, we performed the following experiments. First, co-transfection of a PRMT1 expression construct dose-dependently repressed CIITA-induced MHC II promoter activity in reporter assay (Fig. 3A). Enzyme activity of PRMT1 was clearly required for its ability to repress MHC II transcription by CIITA as an enzyme deficient form of PRMT1 (EQ) failed to impact the MHC II promoter (Fig. 3B). Similarly, we found that PRMT1 over-expression also repressed IFN-γ induced MHC II transactivation in reporter assay (Fig. 3C). Second, PRMT1 silencing by transfection of a short hairpin RNA (shRNA) construct targeting PRMT1 rendered CIITA (Fig. 3D) and IFN-γ (3E) more efficient in activating the MHC II promoter. Finally, small interfering RNA (siRNA) mediated depletion of endogenous PRMT1 (Fig. 3F for knockdown efficiency) resulted in increased synthesis of MHC II (HLA-DRA) molecules in response to IFN-γ stimulation at both message (Fig. 3G), measured by qPCR, and protein (Fig. 3H), measured by flow cytometry, levels. Similar observations that PRMT1 silencing enhanced MHC II stimulation by IFN-γ were made in primary mouse peritoneal macrophages (Fig. 3I–K). Combined, we conclude that PRMT1 could repress MHC II transcription in macrophages.

### PRMT1 methylates and promotes the degradation of CIITA

**Figure 4:**
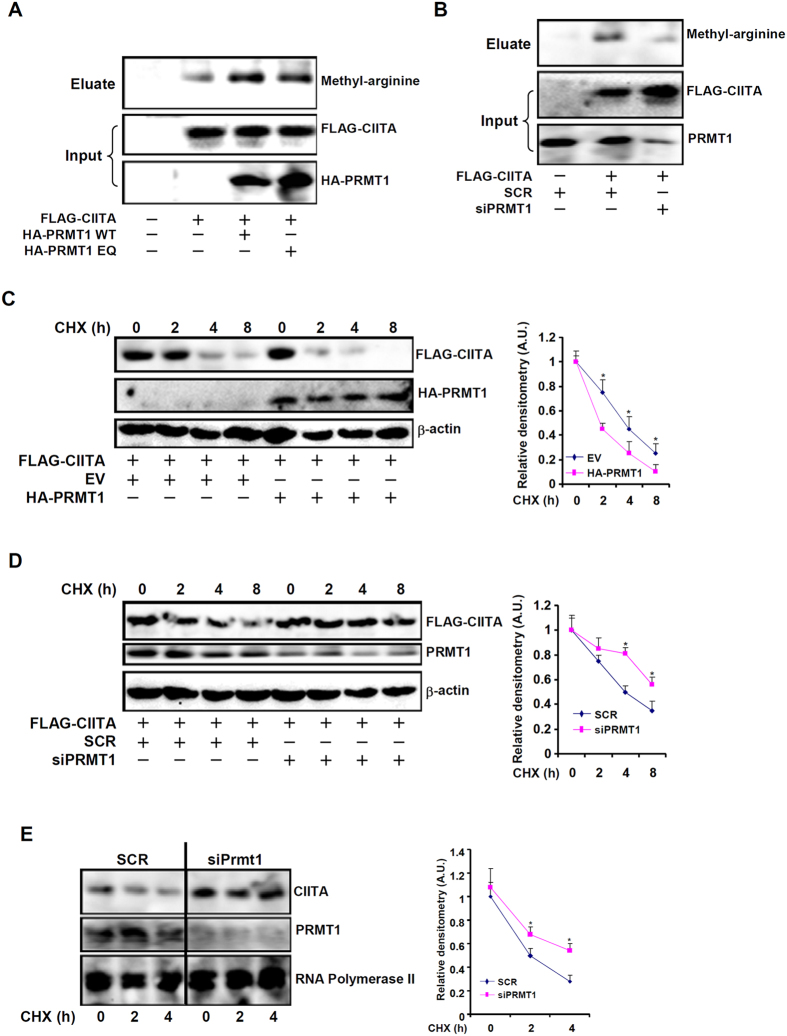
PRMT1 methylates and promotes the degradation of CIITA. (**A**) HEK293 cells were transfected with indicated expression constructs. Immunoprecipitation was performed with anti-FLAG and Western blotting was performed with anti-FLAG, anti-HA, or anti-methylated arginine. (**B**) HEK293 cells were transfected with FLAG-tagged CIITA along with siRNA targeting PRMT1 or a random siRNA (SCR). Immunoprecipitation was performed with anti-FLAG and Western blotting was performed with anti-FLAG, anti-PRMT1, or anti-methylated arginine. (**C**) FLAG-tagged CIITA was transfected into HEK293 cells with HA-tagged PRMT1 or an empty vector (EV). CHX was added 24 hour after transfection and cells were harvested at indicated time points. Western blotting was performed with anti-FLAG, anti-HA, or anti-β-actin. (**D**) FLAG-tagged CIITA was transfected into HEK293 cells with siRNA targeting PRMT1 or a random siRNA (SCR). CHX was added 24 hour after transfection and cells were harvested at indicated time points. Western blotting was performed with anti-FLAG, anti-PRMT1, or anti-β-actin. (**E**) RAW264 cells were transfected with siRNA targeting PRMT1 or a random siRNA (SCR) followed by treatment with IFN-γ. CHX was added 48 hour after transfection and cells were harvested at indicated time points. Western blotting was performed with anti-CIITA, anti-PRMT1, or anti-β-actin.

CIITA activity can be modulated by its post-translational modifications^11^. We postulated that PRMT1 might directly methylate CIITA to influence its activity. Indeed, Western blotting analysis showed that over-expression of wild type PRMT1, but not enzyme deficient PRMT1, increased arginine methylation of CIITA (Fig. 4A). On the contrary, PRMT1 depletion decreased arginine methylation of CIITA (Fig. 4B).

Previously, we have demonstrated that CIITA acetylation affects its protein stability^12^,^13^. Pulse-chase experiments showed that PRMT1 over-expression promoted CIITA degradation (Fig. 4C). On the contrary, PRMT1 depletion prolonged CIITA half-life (Fig. 4D). We also assessed the impact of PRMT1 depletion on the half-life of endogenous CIITA proteins and found that PRMT1 knockdown decelerated degradation of endogenous CIITA in RAW264 cells (Fig. 4E). Thus, we propose that PRMT1 may methylate CIITA to accelerate its degradation.

## Discussion

CIITA dependent MHC II transcription contributes to T lymphocyte activation and adaptive immunity. On the other hand, aberrant MHC II transactivation constitutes a major mechanism underlying chronic inflammation in the vasculature during atherogenesis^10^. We report here that the protein arginine methyltransferase PRMT1 represses MHC II transcription in macrophages by methylating CIITA.

Previous investigations have demonstrated that CIITA activity can be regulated by phosphorylation^18^,^19^, ubiquitination^20^, and acetylation/deacetylation^12^,^13^,^21^. We show here that PRMT1 mediates arginine methylation of CIITA. PRMT1-dependent protein methylation is known to cause a range of different yet not mutually exclusive effects on its targets. For instance, methylation of the p53 binding protein 1 (53BP1) by PRMT1 is necessary for its affinity for DNA and thus essential for DNA repair^22^. Alternatively, methylation of TNF receptor-associated factor 6 (TRAF6) by PRMT1 inhibits the E3 ligase activity of TRAF6 and blocks Toll-like receptor signaling^17^. Our data suggest that PRMT1 promotes CIITA degradation, which is consistent with a previously documented role for PRMT1 in influencing protein turn-over of its targets including Smurf2^23^ and Axin^24^. PRMT1 preferentially methylates arginine residues within the context of the glycine/arginine-rich (GAR) motif although non-GAR substrates have also been identified^25^. At this point, it is not clear what arginine residues within CIITA are targeted by PRMT1. In addition, it remains undetermined whether PRMT1-induced CIITA degradation is solely a result of PRMT1-dependent arginine methylation or a secondary effect contingent on communications with other types of post-translational modifications since PRMT1-dependent arginine methylation has been shown to form crosstalk with acetylation^26^,^27^, ubiquitination^28^,^29^, and phosphorylation^30^,^31^. It has been previously shown that CIITA poly-ubiquitination plays a role in regulating its stability^20^, although CIITA mono-ubiquitination could enhance its activity without altering its half-life^32^. In addition, phosphorylation of CIITA also regulates its stability as shown by Greer and colleagues in a report that demonstrates CIITA Ser280 phosphorylation as part of the degron contributing to its degradation^33^. We have previously shown that deacetylation of CIITA by HDAC2^12^ or SIRT1^13^ could differentially control its stability although the specific lysine residues remain undetermined. More recently, Beaulieu *et al*. have reported that the extreme N-terminus of CIITA is responsible for its rapid degradation: removal of the first 10 amino acids stabilizes CIITA but paradoxically decreases its transcriptional activity^34^. Of note, this part of CIITA contains two arginine residues but it is not clear whether either one could be methylated by PRMT1. Further studies are warranted to solve these issues.

We show here that PRMT1 expression and activity are down-regulated by IFN-γ treatment. Zakrzewicz *et al*. have recently reported that there is elevated expression of PRMT1 in the lungs of patients with idiopathic pulmonary fibrosis or IPF^35^. This finding is in keeping with our observation because we have previously shown that IFN-γ antagonizes the pathogenesis of IPF by inducing CIITA to repress collagen type I transcription^36^,^37^,^38^. Additionally, PRMT1 expression is down-regulated in aging tissues in rats in which there is a concomitant increase of overall inflammation^39^. In light of our finding, it appears that PRMT1 levels/activities may be intimately correlated with tissue inflammation. It is worthwhile to examine PRMT1 levels in the atherosclerotic lesions to verify whether PRMT1 might be down-regulated in the plaque to allow MHC II transactivation during atherogenesis.

Our data suggest that PRMT1 represses MHC II transcription, which could be attributed to CIITA degradation. PRMT1 is found to catalyze asymmetric methylation of histone H4R3, which serves as a permissive step for histone acetylation and transcriptional activation^40^. Therefore PRMT1 could contribute to both transcriptional activation and repression depending on its targets. Future investigations employing both ChIP-sequencing technique and macrophage-specific PRMT1 deletion mouse models will help clarify the role of PRMT1 in transcriptional regulation in the context of atherogenesis *in vivo*.

Several decades of research have unequivocally proven that atherosclerosis is a pathology of chronic inflammation. CIITA-mediated MHC II transactivation in macrophages plays a significant role in the activation of T lymphocytes and therefore vascular inflammation. Our data suggest that PRMT1, by methylating CIITA and promoting CIITA degradation, contributes to the modulation of MHC II trans-activation. Clearly, our data provide rationale for further investigating the role of PRMT1 in vascular inflammation. These efforts aimed at establishing a definitive relationship between PRMT1 and atherogenesis could yield potential interventional strategies in the future.

## Materials and Methods

### Cell culture and treatment

Mouse macrophage cell (RAW264) and human embryonic kidney cell (HEK293) were maintained in DMEM. Mouse primary peritoneal macrophages were isolated as previously described^13^. Mouse recombinant IFN-γ was obtained from R&D and CHX was purchased from Sigma.

### Plasmids, transient transfection, viral Infection, and luciferase assay

FLAG-tagged CIITA, GFP-tagged PRMT1, HA-tagged PRMT1 (WT and EQ), PRMT1 short hairpin RNA (shRNA) plasmid, DRA300 reporter have been previously described^6^,^7^,^14^,^15^,^16^. Small interfering RNA (siRNA) for mouse and human PRMT1 was purchased from Dharmacon. Transient transfections were performed with Lipofectamine 2000 (Invitrogen). Luciferase activities were assayed 24–48 hours after transfection using a luciferase reporter assay system (Promega).

### Protein extraction, immunoprecipitation and Western

Whole cell lysates were obtained by re-suspending cell pellets in RIPA buffer (50 mM Tris pH7.4, 150 mM NaCl, 1% Triton X-100) with freshly added protease inhibitor (Roche). FLAG-conjugated beads (M2, Sigma) were added to and incubated with lysates overnight. Precipitated immune complex was eluted with 3X FLAG peptide (Sigma). Western blot analyses were performed with anti-FLAG, anti-β-actin, anti-HA, anti-GFP (Sigma), anti-CIITA (Santa Cruz), anti-PRMT1, and anti-methylated arginine (Abcam) antibodies.

### Chromatin Immunoprecipitation (ChIP)

ChIP assay was performed essentially as described previously^17^,^18^,^19^,^20^. sChromatin in control and treated cells were cross-linked with 1% formaldehyde. Cells were incubated in lysis buffer (150 mM NaCl, 25 mM Tris pH 7.5, 1% Triton X-100, 0.1% SDS, 0.5% deoxycholate) supplemented with protease inhibitor tablet and PMSF. DNA was fragmented into ∼500 bp pieces using a Branson 250 sonicator. Aliquots of lysates containing 200 μg of protein were used for each immunoprecipitation reaction. Precipitated genomic DNA was amplified by real-time PCR with primers as previously described^41^.

### RNA extraction and real-time PCR

RNA was extracted using an RNeasy RNA isolation kit (Qiagen). Reverse transcriptase reactions were performed using a SuperScript First-strand synthesis system (Invitrogen). Real-time PCR reactions were performed on an ABI STEPONE Plus (Life Tech) using previously described primers^13^.

### Flow cytometry

Cells were incubated with FITC-labeled antibody against H2-IEβ (BD Pharmingen). After PBS washing, labeled cells were detected by a flow cytometer (BD FACSCalibur). Each panel depicts data gathered from 10,000 individual cells. Data are expressed as relative H2-IEb levels compared to the IFN-γ treated group (100%).

### Statistical Analysis

One-way ANOVA with post-hoc Scheffe analyses were performed using an SPSS package. P values smaller than 0.05 were considered statistically significant.

## Additional Information

**How to cite this article:** Fan, Z. *et al*. Protein arginine methyltransferase 1 (PRMT1) represses MHC II transcription in macrophages by methylating CIITA. *Sci. Rep.* **7**, 40531; doi: 10.1038/srep40531 (2017).

**Publisher's note:** Springer Nature remains neutral with regard to jurisdictional claims in published maps and institutional affiliations.
